# Superior Electrochemical Performance of Thin-Film Thermoplastic Elastomer-Coated SnSb as an Anode for Li-ion Batteries

**DOI:** 10.1038/s41598-019-40835-9

**Published:** 2019-03-13

**Authors:** Alexander T. Tesfaye, Frédéric Dumur, Didier Gigmes, Sébastien Maria, Laure Monconduit, Thierry Djenizian

**Affiliations:** 1 Mines Saint-Etienne, Center of Microelectronics in Provence, Department of Flexible Electronics, F – 13541 Gardanne, France; 2 Aix-Marseille University, CNRS, ICR UMR 7273, CROPS, Centre Saint-Jérôme, F-13397 Marseille Cedex 20, France; 3 FR CNRS 3459, Réseau sur le Stockage Electrochimique de l’Energie (RS2E), Paris, France; 4 Institut Charles Gerhardt—Agrégats, Interfaces, Matériaux pour l’Energie, CNRS UMR 5253, Université de Montpellier 2, 34095 Montpellier Cedex 5, France

## Abstract

The high failure strain of thermoplastic elastomers (TPEs) is a very desirable feature for rechargeable Li-ion batteries by improving the lifetime of high specific capacity anode materials that undergo mechanical fractures induced by large volume variations. In this work, poly(styrene-*b*-2-hydroxyethyl acrylate) called PS-*b*-PHEA was synthesized by a nitroxide meditated polymerization method. Owing to the use of a specific polystyrene macroinitiator (SG1), a suitable TPE copolymer with long hydroxyethyl acrylate blocks to ensure good mechanical properties is obtained for the first time. We show that the electrochemical properties of the PS-*b*-PHEA-coated SnSb anode are drastically improved by suppressing the crack formation at the surface of the electrode. Indeed, electrochemical characterization revealed that a high and stable gravimetric capacity over 100 cycles could be achieved. Moreover, excellent capacity reversibility was achieved when cycled at multiple C-rates and fast kinetics confirming the strong protection role of the polymer. The advanced chemical and mechanical properties of PS-*b*-PHEA open up promising perspectives to significantly improve the electrochemical performance of all electrodes that are known to suffer from large volume variations.

## Introduction

There is great interest in developing rechargeable Li-ion batteries (LIBs) with high energy density and long cycle life for a wide variety of applications such as portable electronic devices, electric and hybrid vehicles, and grid-scale energy storage systems^1–4^. Currently, commercialized LIBs use carbonaceous materials as the negative electrode, however, the technology reaches a limit because of the low specific capacity (372 mAh g^−1^) corresponding with the formation of LiC_6_^5^. Antimony and tin have long been considered as an attractive replacement for graphite electrode in LIB systems due to their superior lithium storage capacities of 660 and 994 mAh g^−1^, respectively^5–8^. Unfortunately, the electrochemical performance of these materials fades during cycling because of the electrical contact loss induced by large volume variations combined with the continuous growth of a solid electrolyte interphase (SEI) layer at the surface hindering the electron transfer^9^. The use of multi-phase active materials, such as SnSb, instead of pure metals (Sn or Sb) has been used to limit the large volume change by reacting with Li^+^ at different stages during the charge and discharge process^10,11^. SnSb reacts reversibly with Li^+^ by an alloying/dealloying mechanism according to Equations (1) and (2)^9,11–13^.

1 $$SnSb+3L{i}^{+}+3{e}^{-}\leftrightarrow L{i}_{3}Snb+Sn$$

2 $$Sn+xL{i}^{+}+x{e}^{-}\leftrightarrow L{i}_{x}Sn\,\,\,\,\,\,\,\,0 < x < 4.4$$

Unfortunately, the alloying/dealloying reaction is still accompanied by a volume change which results in the loss of electrical contact with the active material, Sn agglomeration, and the formation of SEI film on the electrode surface leading to poor electrochemical performance^14–16^. Much effort has been devoted to improving the cycling performance, which includes chemical modification of the electrolyte by adding various additives like vinylene carbonate (VC) and fluoroethylene carbonate (FEC) to create a thin stable protective layer^12,17^. It has also been reported that the electrochemical properties of Si and TiO_2_ can be improved by coating the electrode surface with inorganic or organic compounds, such as Ni, Al_2_O_3_, self-healing polymer, and polyaniline^18–20^. In the present work, the latter approach is investigated by coating micron-sized SnSb with a new thermoplastic elastomer (TPE) thin layer. Typically, TPEs are nanostructured materials based on block (or graft) copolymers with short hard blocks and long soft blocks^21^. The glassy nanodomains dispersed in the rubbery matrix ensure rubber elasticity thanks to reversible physical crosslinking and give mechanical resistance to the TPEs^22^. In order to prevent mechanical damage, an elastomer showing high strain property and good adhesion to the surface of the active material has been designed. The synthesis of a block copolymer poly(styrene-*b*-2-hydroxyethyl acrylate) so-called PS-*b*-PHEA is studied using polystyrene (PS) as the hard block and PHEA as the soft block, which is rubbery at room temperature and in addition known for its biocompatibility and lack of toxicity^23^. Moreover, the block copolymer is expected to have a good affinity for the electrode thanks to hydrogen bonds formed between the hydroxyl groups present as pending functions on PHEA molecules and terminated groups at the surface of SnSb particles. In this work, the synthesis of the targeted block copolymer PS-*b*-PHEA is investigated by nitroxide mediated polymerization (NMP), which is a simple method of controlled radical polymerization (CRP) allowing the tuning of the mechanical properties of the copolymer through precise control of the molar mass and dispersity of both the hard and soft blocks^24^. Compared to other synthesis methods, NMP is an unimolecular technique involving only one alkoxyamine initiator with no additional controller and no purification step^25,26^. Recently, PS-*b*-PHEA could be synthetized by different CRP methods including NMP with TEMPO-and TIPNO-based nitroxide^27–31^. But to the best of our knowledge, only short PHEA blocks not exceeding 50 wt. % of the copolymer have been reported limiting the deformation property.

For the first time, we report that PS-*b*-PHEA carrying long PHEA blocks can be obtained by NMP controlled with SG1 nitroxide. We also show that a PS-*b*-PHEA thin layer deposited onto micron-sized SnSb can stabilize the electrode/electrolyte interface, leading to significant electrochemical performance.

## Results and Discussion

### Material Synthesis

**Figure 1:**
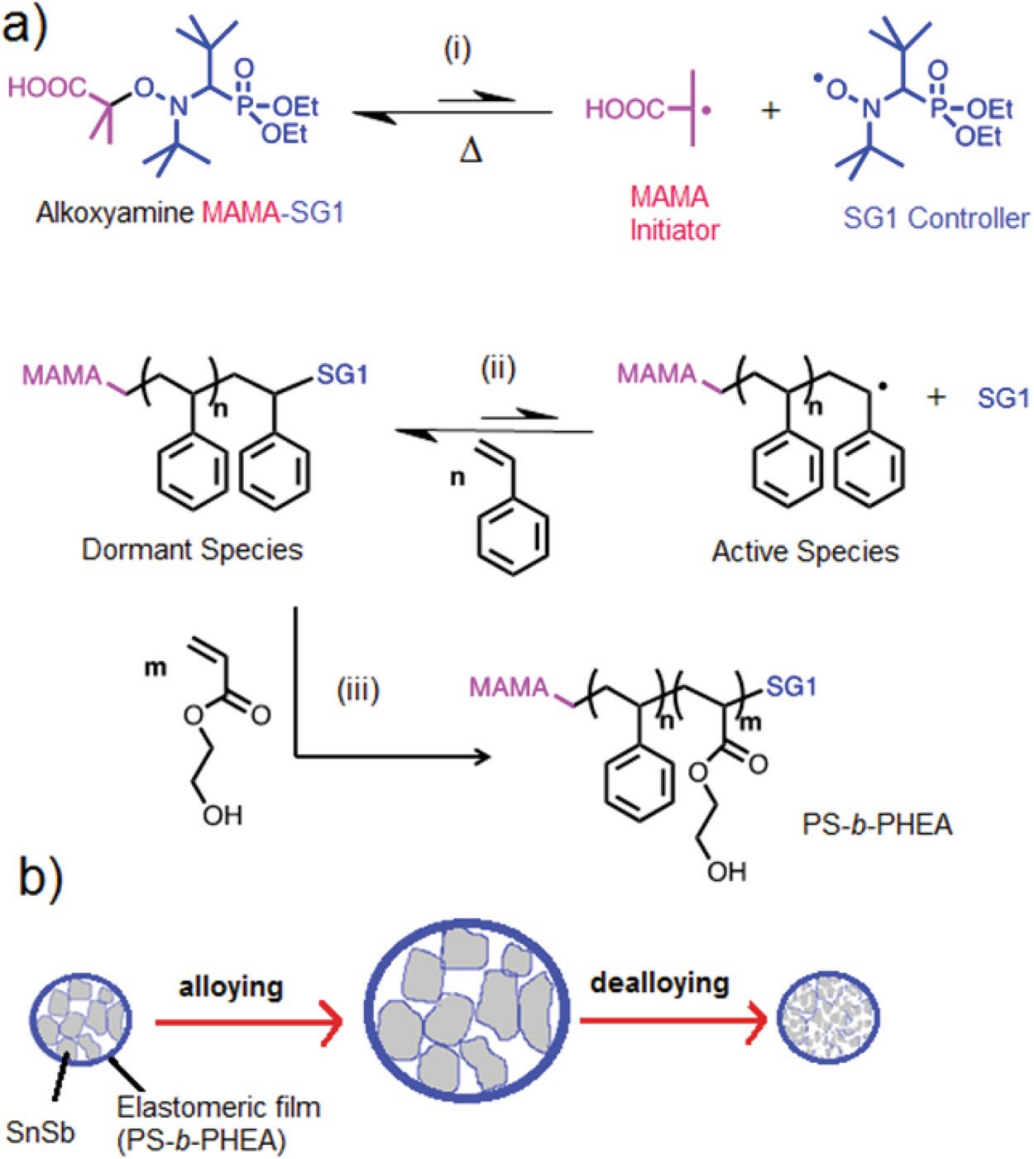
(**a**) The synthesis mechanism of PS-*b*-PHEA using NMP: (i) Initiation of radicals, (ii) NMP mechanism with styrene, and (iii) synthesis of block copolymer PS-*b*-PHEA. (**b**) Schematic representation of the stretchable behaviour of the PS-*b*-PHEA elastomer coating during alloying/de-alloying mechanism of SnSb particles.

Figure 1a describes the synthesis of PS-*b*-PHEA. In the first step, Fig.1a (i and ii), polystyrene PS-SG1 with low molecular weight and dispersity (*M*_n_ = 6500 g mol^−1^ and *Đ* = 1.13) was synthetized in bulk thanks to the presence of MAMA-SG1 as an initiator/ controlling agent at 120 °C. Then, a reversible termination reaction is induced where propagating macro-radicals react reversibly with stable nitroxides SG1 to give macroalkoxyamine dormant species, Fig. 1a(ii). This equilibrium is fast, allowing the controlled addition of monomers, and shifted toward the dormant species, diminishing the radical concentration and thus the probability of a bimolecular termination reaction. Figure 1a(iii) shows the use of PS-SG1 as alkoxyamine macroinitiator to perform the controlled polymerization of HEA in DMF at 120 °C. A small amount of free nitroxide SG1 was also initially added, since it is known to better control the polymerization of acrylates^32^. Indeed, acrylates have a high propagation rate constant and the complete monomers conversion can occur before enough nitroxides are generated to establish the control equilibrium.

**Figure 2:**
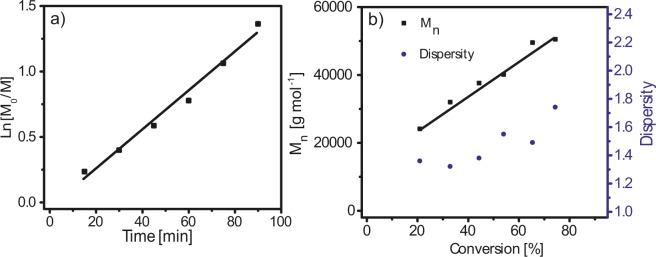
(**a**) Variation of ln[Mo/M] vs. time and (**b**) average number molar mass *M*_*n*_ and dispersity *Đ* vs. HEA conversion for PS-*b*-PHEA synthesized by NMP.

The study of kinetics was carried out to show that the NMP polymerization of HEA from PS- SG1 occurred in a well-controlled manner. As shown in Fig. 2a, ln([M]_o_/[M]) vs. time is linear suggesting that the concentration of propagating radicals remains constant in the course of the polymerization, indicating that a sufficient amount of free nitroxide was initially added, and therefore the undesired bimolecular termination reactions should be negligible. Indeed, as shown in Fig. 2b, the average molar mass of the copolymer increased linearly with the conversion and the molar mass distributions stay low with dispersity values below 1.5 until 65% conversion. This result is good considering that the polymerization occurs from a PS macroinitiator and that a high molar mass was targeted, i.e. 70,000 g mol^−1^ at 100% conversion. The desired PS-*b*-PHEA copolymer revealed a PS weight fraction of 10% (determined by ^1^H NMR), which represents the suitable proportion for producing organized PS nanospheres in the PHEA matrix.

The average number molar mass *M*_n_ obtained by size exclusion chromatography (SEC) was 65,200 g mol^−1^, which is higher than the value of 55,000 g mol^−1^ calculated from the conversion of 82% measured by ^1^H NMR, but SEC molar masses are measured relative to PMMA standards. The copolymer exhibits a dispersity *Đ* of 1.91, which is quite large but the conversion and the PHEA molar mass were high.

**Figure 3:**
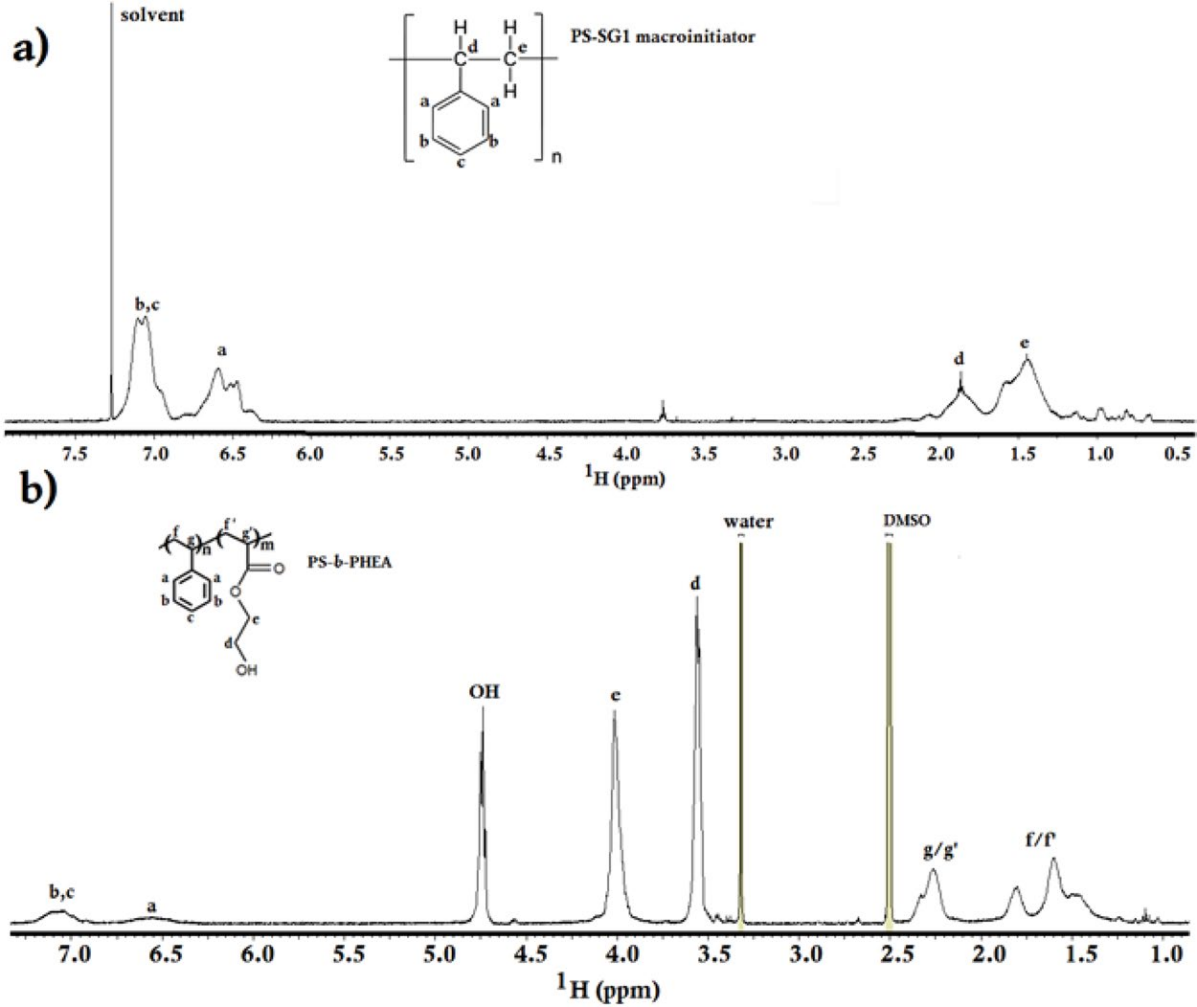
^1^H NMR spectrum of (**a**) PS-SG1 macroinitiator in CDCl_3_ and (**b**) PS-*b*-PHEA in DMSO-d_6_.

The chemical structure and composition of the block copolymer was investigated by ^1^H NMR spectroscopy and FTIR spectroscopy. Figure 3a shows the ^1^H NMR spectrum of the PS-SG1 macroinitiator. Signals a and (b, c) at 7.00 and 6.6 ppm, respectively, are attributed to the phenyl rings of PS. Signals d and e at 1.8 and 1.4 ppm are ascribed to the aliphatic (–CH) and (–CH_2_) protons of the PS chain, respectively. The ^1^H NMR spectrum obtained for PS-*b*-PHEA is shown in Fig. 3b. The signals at 6.50–7.30 ppm are related to the protons of the aromatic styrene groups (a,b,c). The strong signals at 3.55 and 4.05 ppm are attributed to the methylene (–CH_2_) protons of the hydroxyethyl group (d and e) and the signal at 4.8 ppm corresponds to the –OH of hydroxyethyl group. The presence of peaks belonging to styrene and HEA are consistent with the literature data reported for the copolymerization of PS-*b*-PHEA^27,29^.

**Figure 4:**
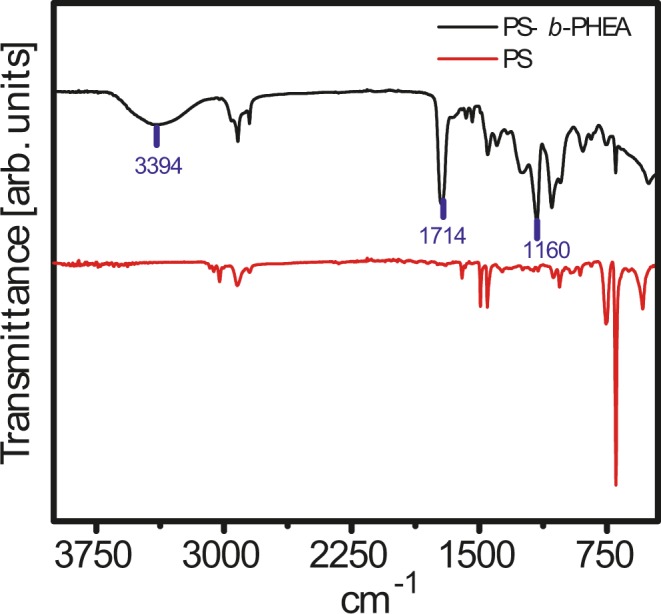
FTIR spectra of PS and PS-*b*-PHEA.

FTIR spectroscopy analysis of PS and PS-*b*-PHEA are shown in Fig. 4. In the FTIR spectrum of PS the aromatic (C=C stretching) band produced three peaks at 1602, 1492, and 1451 cm^−1^, the aromatic (C–H) bands was observed at 740 cm^−1^, and aliphatic band (C–H stretching) appeared at 3024 cm^−1^. The FTIR spectrum of PS-*b*-PHEA exhibits new stretching bands at 3432, 1714, and 1160 cm^−1^, which are assigned to (–OH), –C=O, and C–O of PHEA, respectively^27,33^. These FTIR spectroscopy results are clear evidence of the copolymerization of styrene and HEA by NMP.

### Electrochemical Characterization

**Figure 5:**
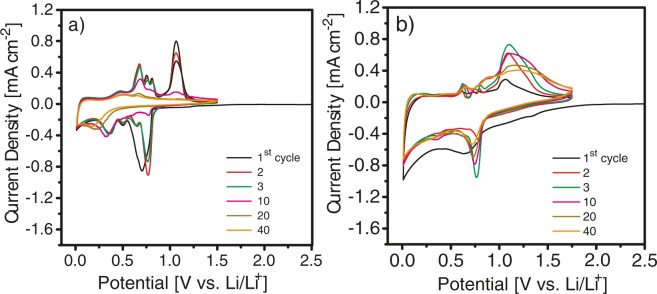
Cyclic voltammograms of (**a**) as-prepared SnSb and (**b**) PS-*b*-PHEA-coated SnSb recorded at a scan rate of 0.1 mV s^−1^ in the potential window of 0.01 V–1.75 V vs. Li/Li^+^.

Figure 5a and b shows curves obtained by cyclic voltammetry (CV) recorded up to the 40^th^ cycle for SnSb electrodes before and after PS-*b*-PHEA coating, respectively. In agreement with previous studies, the electrochemical reactivity for both electrodes is confirmed by the presence of five cathodic peaks during the first cycle. The broad peak around 0.8 V vs. Li/Li^+^ corresponds to the alloying of Li^+^ with SnSb to form Li_3_Sb and Sn and the formation of the SEI film at the electrode surface^34,35^. The two peaks at 0.5 V and 0.6 V vs. Li/Li^+^ are attributed to the lithiation of Sn to form Li_x_Sn. Further alloying of Sn occurs between 0.3 – 0.1 V vs. Li/Li^+ ^^34–36^. Five distinct peaks were obtained during the first anodic scan. The peaks at 0.45 V, 0.7 V, 0.75 V, and 0.8 V vs. Li/Li^+^ are assigned to the delithiation of Li_x_Sn alloy to form Sn. The peak at 1 V vs. Li/Li^+^ is ascribed to the dealloying of Sb to form again SnSb^37^. Compared to the CV curve of as-prepared SnSb electrode, the PS-*b*-PHEA-coated SnSb shows broader peaks and larger surface area under the CV curve. This effect could be attributed to an improved structural and chemical stability of the polymer coated SnSb electrode.

For both electrodes, the anodic/cathodic peaks are reversibly obtained for the first five cycles. After the 5^th^ cycle, the peak intensity corresponding to Li^+^ reaction with Sb is diminishing for the as-prepared SnSb electrode (Fig. 5a). In our previous paper we report the poor electrochemical performance of SnSb is attributed to the pulverization of the active material and the loss of electrical contact between active particles, conductive additives and the current collector as the result of a large volume change during the alloying/dealloying mechanism in addition to the formation and growth of the SEI film^16^. In contrast, reversible alloying/dealloying peaks for PS-*b*-PHEA-coated SnSb were observed up to 40 cycles (Fig. 5b). Moreover, the characteristic of the initial CV for PS-*b*-PHEA-coated SnSb is different from the subsequent CVs as oxidation/reduction peaks tend to enlarge. This effect can be explained by the creation of new active surfaces during the successive alloying/dealloying reactions. The stability of the redox peaks is attributed to the elastomer polymer film at the surface of SnSb. Indeed, highly stretchable PS-*b*-PHEA is able to bear the mechanical stress caused by the large volume variations and thus maintains good quality at the electrode/electrolyte interface and the inter-grains electronic percolation pathways during successive charging and discharging of the battery as depicted in Fig. 1b. The elastomeric property of PS-*b*-PHEA was confirmed by a tensile stress test (see Supplementary Fig. S2). The polymer can be easily stretched up to 200% and reveals a very low value Young’s modulus of 0.01 MPa, which is typical for TPEs with a tensile strength of 0.8 MPa^38–40^. Additionally, the mechanical test also shows that PS-*b*-PHEA can be elongated up to 400% without rupture.

**Figure 6:**
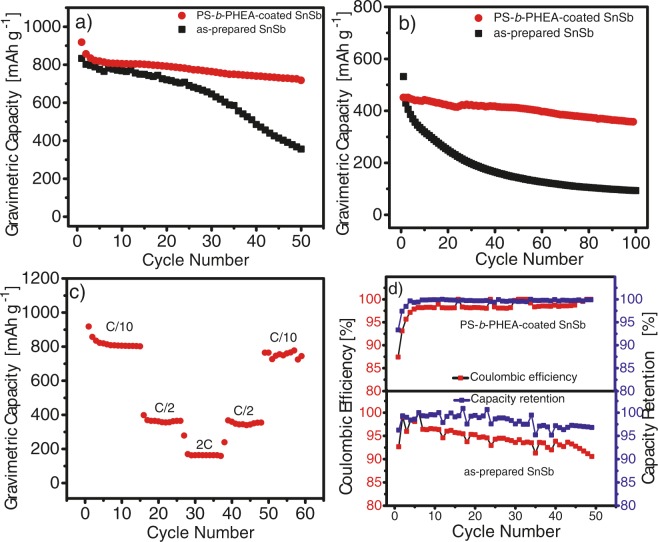
Cycling stability study showing the variation of the discharge capacity versus the cycle number for PS-*b*-PHEA-coated SnSb and as-prepared SnSb at a rate of (**a**) C/10 over 50 cycles and (**b**) 1 C over 100 cycles, (**c**) cycling performance of PS-*b*-PHEA-coated SnSb at multiple C-rates, (**d**) coulombic efficiency and capacity retention vs. cycle number at a rate of C/10 for PS-*b*-PHEA-coated SnSb and as-prepared SnSb.

The electrochemical performance of PS-*b*-PHEA-coated SnSb was evaluated through the examination of the charge/discharge profiles obtained by galvanostatic cycling tests. Figure 6a shows the discharge capacity vs. cycle number for the as-prepared SnSb and the polymer-coated SnSb cycled at C/10 (i.e. 0.0827 A g^−1^). It is clearly apparent that the PS-*b*-PHEA-coated SnSb has superior cyclability than the non-coated SnSb with a reversible capacity of 720 mAh g^−1^ obtained, whereas only 356 mAh g^−1^ was retained after 50 cycles for the as-prepared SnSb electrode. The remarkable stable capacity is attributed to the presence of PS-*b*-PHEA film conferring to SnSb enhanced mechanical and chemical properties. Actually, the copolymer is able to limit the formation of fracture as well as the continuous growth of the SEI. The beneficial effect of the polymer layer is also evidenced at faster kinetics (1 C) over 100 cycles (Fig. 6b). Indeed, the polymer-protected electrode is able to maintain a capacity of 308 mAh g^−1^, whereas the as-prepared SnSb retains only 93 mAh g^−1^.

To study the operational stability of PS-*b*-PHEA-coated SnSb, the cycle life performance has been studied at multiple C-rates (Fig. 6c). A stable gravimetric capacity of 800 mAh g^−1^, 368 mAh g^−1^, and 170 mAh g^−1^ were obtained at C/10 (0.0827 Ah g^−1^), C/2 (0.4135 Ah g^−1^), and 2 C (1.654 Ah g^−1^) rates, respectively. When the rates was subsequently restored to C/2 and C/10, capacity values of 344 mAh g^−1^ and 770 mAh g^−1^ were obtained respectively. The excellent rate capability is again attributed to the PS-*b*-PHEA film, which enables the Li^+^ transfer while ensuring the mechanical stability of the electrode.

Coulombic efficiency (CE) and capacity retention are important criteria for evaluating the electrochemical performance of electrodes for LIBs^16,18,41,42^. The CE obtained for PS-*b*-PHEA-coated SnSb at the first cycle was 87% and reached more than 98% while discharge capacity retention of more than 95% was obtained after 50 cycles, for the as-prepared SnSb a CE of 92% and 90% obtained after the first and 50 cycles (Fig. 6d). These values likely indicate relatively more stable SEI formation on the surface of the polymer-covered electrode even after long term cycling.

**Figure 7:**
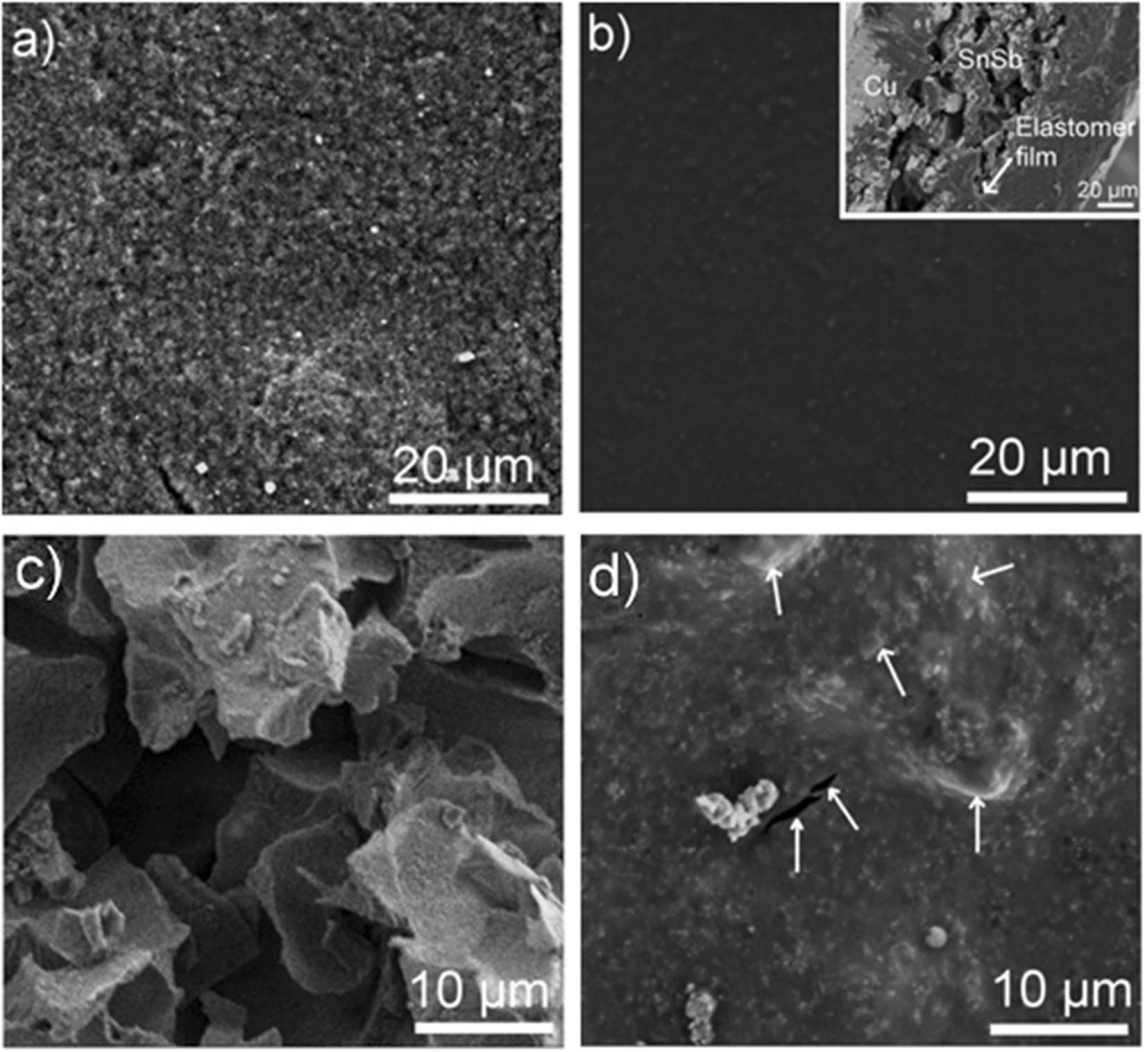
SEM images of (**a**) as-prepared SnSb and (**b**) PS-*b*-PHEA-coated SnSb before cycling. Inset: cross-sectional view. SEM images of (**c**) as-prepared SnSb and (**d**) PS-*b*-PHEA-coated SnSb after 50 cycles at C/10. Minor cracks and lumps are shown by arrows.

To provide further evidence for the positive contribution of the elastomer film on the electrochemical performances, SEM analysis was carried out before and after cycling tests. Figure 7a and b show the SEM top-view images of the as-prepared SnSb and PS-*b*-PHEA-coated SnSb before electrochemical tests, respectively. Compared to the morphological features of as-prepared SnSb, the polymer-covered electrode is characterized by a crack-free and smooth surface. The inset shows that the thermoplastic elastomer film has a thickness of ~2 µm. After 50 charge/discharge experiments performed at a rate of C/10, the surface of the non-coated SnSb electrode is characterized by the presence of large cracks and pits containing different particle sizes (Fig. 7c). This is due to the repeated volume change, which results in the pulverization of the SnSb particles, and thus poor cycling stability (Fig. 6a). For comparison, the surface of PS-*b*-PHEA-coated SnSb electrode is almost unchanged (Fig. 7d). It can be simply noted the apparition of a few small cracks and lumps. Thus, no significant modification of the surface nor delamination were observed thanks to the highly rubbery nature of the polymer coating, which mitigates the cyclic volume variations.

## Conclusion

In this work, enhanced electrochemical performance of SnSb can be achieved by coating the surface with a new TPE. Highly rubbery PS-*b*-PHEA was synthesized by the NMP method using a specific polystyrene macroinitiator allowing the presence of long hydroxyethyl acrylate blocks. The excellent cycle life performance and rate capability of PS-*b*-PHEA-coated SnSb obtained is attributed to the high failure strain characteristics of the elastomer film. The copolymer can strongly mitigate the volume expansion/contraction during Li^+^ alloying/dealloying reactions with SnSb and the SEI formation. As a result, the SnSb electrode is chemically and mechanically stable beyond 100 cycles even at fast kinetics. Moreover, the use of the PS-*b*-PHEA coating can extend to other high capacity materials like silicon and conversion electrodes that suffer from large volume changes during cycling.

## Methods

### Material Synthesis and Instrumentation

Tetrahydrofurane (THF), N,N-Dimethylformamide (DMF), Methanol, 2-hydroxyethyl acrylate (HEA) and styrene (S) were purchased from Sigma-Aldrich and were used as received. (*N*-*tert*-butyl-*N*-(1′-diethylphosphono-2,2′-dimethylpropyl)-*O*-(2-carboxyl-prop-2-yl) (MAMA-SG1) alkoxyamine initiator (registered as trademark BlocBuilder by Arkema) and *N*-*tert*-butyl-*N*-(1′-diethylphosphono-2,2′-dimethylpropyl) (SG1, 85%) free nitroxide were provided by Arkema and were used as received.

The chemical structure and composition of the block copolymer was investigated by proton nuclear magnetic resonance spectroscopy (^1^H NMR) and Fourier Transform Infrared (FTIR) spectroscopy. NMR experiments were performed with a Bruker Avance III 400 MHz Nanobay spectrometer. ^1^H NMR spectra were recorded at a frequency of 400 MHz with a 11.3 μs 30° pulse, a repetition time of 4 s and 16 scans. NMR chemical shifts were reported in standard format as values in ppm relative to deuterated solvents. Infrared analysis was carried out using a Perkin Elmer Spectrum 2 equipped with a single reflection diamond module (ATR). IR spectrum was recorded in the 400–4000 cm^−1^ range, at 4 cm^−1^ resolution.

### Synthesis of Macroinitiator, PS-SG1

MAMA-SG1 (0.768 g, 25.6 mmol) and styrene (20 g, 192 mmol, 95 eq.) were placed in a three-neck round-bottom flask equipped with a reflux condenser and a magnetic stir bar. The mixture was purged for 30 min with argon to remove oxygen. The mixture was immersed in an oil bath and heated to 120 °C (in the solution) with a 30 min ramp temperature. After 2 h, cooling down the mixture in an ice bath stopped the polymerization. The crude product was diluted in THF (20 mL) and then poured into a large excess of cold ethanol. The solid was collected by filtration, re-dissolved in THF, and re-precipitated in cold methanol. Then, the precipitate was dried under vacuum (5 × 10^−2^ mbar) after filtration. A *M*_n_ of 6500 g mol^−1^ with a *Đ* of 1.13 were determined by size exclusion chromatography (SEC) carried out in THF at 70 °C relative to PS standards (see Supplementary Fig. S1).

### Synthesis of Poly(styrene-b-2-hydroxyethyl acrylate) Block Copolymer, PS-b-PHEA

PS-SG1 macroinitiator (0.4418 g, 0.068 mmol) and SG1 free nitroxide (1.98 mg, 0.00674 mmol, 0.1 eq.), dissolved in DMF (4 g), were placed in a three-neck round-bottom flask equipped with a reflux condenser and a magnetic stir bar. Then HEA (4.002 g, 34.5 mmol, 507 eq.) were added and the solution was purged for 30 min with argon to remove oxygen. The mixture was immersed in an oil bath and heated to 120 °C (in the solution) with a 20 min temperature ramp. Aliquots were withdrawn periodically for monitoring conversions by ^1^H NMR and molar masses by SEC. After 90 min, cooling down the mixture in an ice bath stopped the polymerization. The final conversion was 82%. The crude product was diluted in THF (15 mL) and then poured into a large excess of cold diethyl ether. The precipitated viscous polymer stuck on the bottom of the beaker and was collected by decanting the solvent in the fridge, followed by washing 3 times with diethyl ether and drying under reduced pressure. The solid was re-dissolved in DMF and then re-precipitated in cold diethyl ether. Finally, the solvent was decanted in the fridge, followed by washing 3 times with diethyl ether, and drying under reduced pressure, yielding the desired copolymer with *M*_n_ = 65,200 g mol^−1^ and *Đ* = 1.91 (determined by SEC in DMF at 70 °C relative to PMMA standards, see Fig. S1).

### Electrode Fabrication

PS-*b*-PHEA was dissolved in DMF (1.5 mL) and then mixed with Carbon black SP (Alfa Aesar) and Li-salt bis(trifluoromethane)sulfonimide-LiTFSi (Sigma Aldrich) with a mass ratio of 70:15:15 using magnetic stirrer. The homogenous suspension was then drop-casted onto Teflon and dried overnight at 80 °C to form a conductive elastomer thin film. Crystallized SnSb powder up to 1 g was obtained using a very simple dry microwave route under one minute and a half ^8^. The SnSb electrode was fabricated using a slurry cast method. First, SnSb powder was mixed with polyvinylidene fluoride (PVDF) as a binding agent, and Carbon black SP as the conducting agent with a mass ratio of 70:15:15 in vibrational ball milling for 25 min at 20 Hz. Then, the slurry was tape casted on a Cu current collector at room temperature for 24 h and then further dried at 110 °C for 12 h under vacuum. Then, the SnSb electrode was heated to 90 °C on a hot plate. The conductive composite copolymer film was then melted at 100 °C and coated on the SnSb electrode with a sharp blade. The electrodes were degassed in vacuum at room temperature overnight and transferred to an argon glove box for the battery assembly.

Standard two-electrode Swagelok cells were assembled in a glove box filled with high purity argon. The half-cells consisted of PS-*b*-PHEA-coated SnSb as the working electrode and a battery grade Li foil as the counter electrode. The two electrodes were separated by a Whatman glass microfiber soaked in the liquid electrolyte: 1 M LiPF_6_ in ethylene carbonate, propylene carbonate, and dimethyl carbonate (1:1:3 volume ratio) with 1 vol.% VC and 5 vol.% FEC. The electrochemical characterization was performed by cyclic voltammetry (CV) and chronopotentiometry using a VMP3 potentiostat (Bio Logic, France). The CV was performed in the potential window of 0.01–1.75 V vs. Li/Li^+^ at a scan rate of 0.1 mV s^−1^. The galvanostatic tests were done at various C-rates, where C/n means the battery is fully charged or discharged up to its total storage capacity in n hours (for this work 1 C=0.827 A g^−1^).

The morphology of PS-*b*-PHEA-coated SnSb before and after cycling experiments was examined by a field-emission scanning electron microscope (FE-SEM Ultra-55 Carl Zeiss).

## Supplementary information

Dataset1
