# Supplementary material for: Superior Electrochemical Performance of Thin-Film Thermoplastic Elastomer-Coated SnSb as an Anode for Li-ion Batteries
Source: Sci Rep. 2019 Mar 13;9:4301. doi: 10.1038/s41598-019-40835-9 (PMC6416307; doi:10.1038/s41598-019-40835-9)
Supplement: Supplementary file 1 — Dataset1 [file 41598_2019_40835_MOESM1_ESM.docx]

Supplementary data

Superior Electrochemical Performance of Thin-Film Thermoplastic Elastomer-Coated SnSb as an Anode for Li-ion Batteries

Alexander T. Tesfaye^1^, Frédéric Dumur^2,3^, Didier Gigmes^2,3^, Sébastien Maria^2,3^, Laure Monconduit^3,4^, and Thierry Djenizian^1,^*

^1^Mines Saint-Etienne, Center of Microelectronics in Provence, Department of Flexible Electronics, F – 13541 Gardanne, France

^2^Aix-Marseille University, CNRS, ICR UMR 7273, CROPS, Centre Saint-Jérôme, F-13397 Marseille Cedex 20, France

^3^FR CNRS 3459, Réseau sur le Stockage Electrochimique de l’Energie (RS2E), Paris,

France

^4^Institut Charles Gerhardt — Agrégats, Interfaces, Matériaux pour l'Energie, CNRS UMR 5253, Université de Montpellier 2, 34095 Montpellier Cedex 5, France

*E-mail: [thierry.djenizian@emse.fr](mailto:thierry.djenizian@emse.fr)

1. Molar mass determination by size exclusion chromatography

Polymer molar masses and dispersities were determined by size exclusion chromatography. (SEC). The SEC experiments for PS-SG1 macroinitiator were performed on an EcoSEC apparatus from PSS, equipped with a dual flow cell refractive index detector. Eluent was THF at a flow rate of 0.3 mL.min-1 for the sample pump and 0.15 mL.min-1 for the reference pump. The stationary phase was a combination of one PL Resipore (50x4.6) mm guard column and two PL Resipore (250x4.6) mm columns thermostated at 40°C. Samples were prepared at concentration of 0.25 wt.% in THF containing 0.25 vol.% of toluene, as a flowmarker. Injection volume was 20 μL. Polystyrene equivalent number-average molar masses (*M*_n_) and dispersities *Ɖ* were calculated by means of PS calibration curve using PS-M Easivial standards (Agilent, USA). SEC experiments for PS-b-PHEA were carried out on the following columns were used: one pre-column and two PL Resipore columns. The injection loop, the columns and the RI detector were in the same oven thermostated at 70°C. The eluent was a solution of 0.1 M LiBr in dimethylformamide and the flow rate was fixed at 0.7 mL min−1. The samples were prepared in a mixture of eluent and toluene (0.25 vol%) as flowmarker, filtered through a 0.2 μm Nylon filter (Interchim) and placed in an auto-sampler preheated at 50°C. Samples concentration was 0.25 wt%. Calibration curves were established with poly(methyl methacrylate) (PMMA) standards purchased from PSS polymers


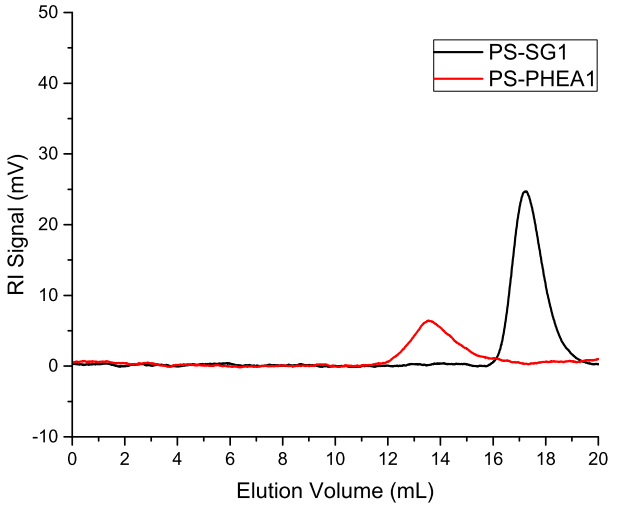


Supplementary Figure S1. Molecular weight distribution of macroinitiator PS-SG1 and PS-*b*-PHEA.

Supplementary Table S1. Superposition of macroinitiator PS-SG1 and PS-*b*-PHEA chromatograms obtained by SEC in DMF.

|  | M_n_ (g mol^–1^) | M_w_ (g mol^–1^) | *Đ* | M_p_ (g mol^–1^) |
| --- | --- | --- | --- | --- |
| PS-SG1 | 6496 | 7351 | 1.13 | 7161 |
| PS-*b*-PHEA | 65200 | 78700 | 1.91 | 76700 |

Where: Mn= Number-average molecular weight, Mw = Weight-average molecular weight, *Đ*= Dispersity

1. Mechanical properties of PS-*b*-PHEA

The mechanical property of PS-*b*-PHEA was investigated using tensile stress test. Figure S2 shows stress-strain curves obtained for PS-*b*-PHEA recorded at strain rate of 5 mm min^–1^ at 25 ^0^C. The result shows the rupture strain over 400 % of the original length. The stress-strain curve characteristic of PS-*b*-PHEA is typical of the thermoplastic elastomer polymers.


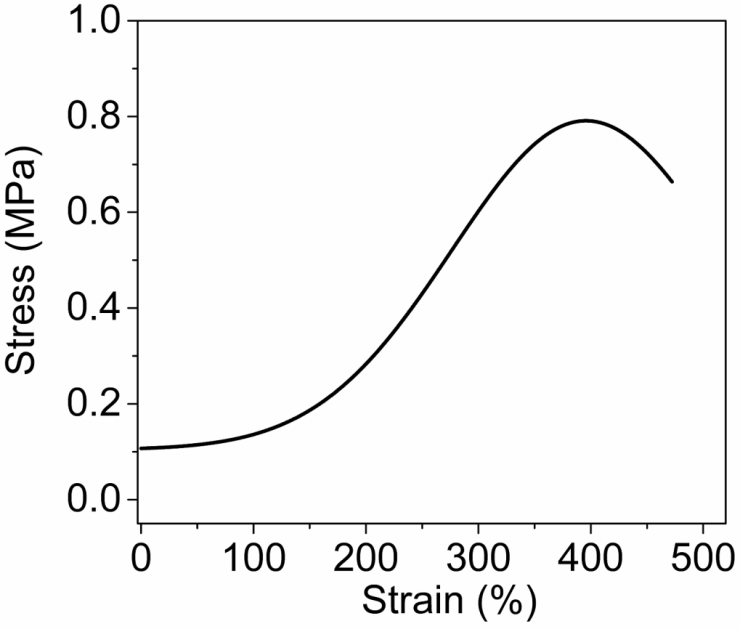


Supplementary Figure S2. Stress-strain curve obtained for PS-*b*-PHEA at strain rate of 5 mm min^–1^.
